# Household expenditure for immunization among children in India: a two-part model approach

**DOI:** 10.1186/s12913-021-07011-0

**Published:** 2021-09-22

**Authors:** Shobhit Srivastava, Pradeep Kumar, Shekhar Chauhan, Adrita Banerjee

**Affiliations:** 1 Department of Mathematical Demography & Statistics, International Institute for Population Sciences, Mumbai, India; 2 Department of Population Policies and Programmes, International Institute for Population Sciences, Mumbai, India; 3 Department of Public Health and Mortality Studies, International Institute for Population Sciences, Mumbai, India

**Keywords:** Immunization, Expenditure, Two-part model, NSSO, India

## Abstract

**Background:**

Despite the Indian government’s Universal Immunization Program (UIP), the progress of full immunization coverage is plodding. The cost of delivering routine immunization varies widely across facilities within country and across country. However, the cost an individual bears on child immunization has not been focussed. In this context, this study tries to estimate the expenditure on immunization which an individual bears and the factors affecting immunization coverage at the regional level.

**Methods:**

Using the 75th round of National Sample Survey Organization data, the present paper attempts to check the individual expenditure on immunization and the factors affecting immunization coverage at the regional level. Descriptive statistics and multivariate regression analysis were used to fulfil the study objectives. The two-part model has been employed to inspect the determinants of expenditure on immunization.

**Results:**

The overall prevalence of full immunization was 59.3 % in India. Full immunization was highest in Manipur (75.2 %) and lowest in Nagaland (12.8 %). The mean expenditure incurred on immunization varies from as low as Rs. 32.7 in Tripura to as high as Rs. 1008 in Delhi. Children belonging to the urban area [OR: 1.04; CI: 1.035, 1.037] and richer wealth quintile [OR: 1.14; CI: 1.134–1.137] had higher odds of getting immunization. Moreover, expenditure on immunization was high among children from the urban area [Rs. 273], rich wealth quintile [Rs. 297] and who got immunized in a private facility [Rs. 1656].

**Conclusions:**

There exists regional inequality in immunization coverage as well as in expenditure incurred on immunization. Based on the findings, we suggest looking for the supply through follow-up and demand through spreading awareness through mass media for immunization.

**Supplementary Information:**

The online version contains supplementary material available at 10.1186/s12913-021-07011-0.

## Background

Vaccinations are one of the most cost-effective and impactful health interventions used worldwide and have resulted in dramatic declines and regional elimination of many serious childhood infectious diseases [1]. The World Health Organization (WHO) estimates that about 2–3 million deaths under 5 years of age could be preventable through immunization [2]. Nonetheless, the WHO estimates that vaccine-preventable deaths (VPD’s) are still responsible for 1.5 million deaths each year [2]. The recent estimates on immunization coverage by WHO and UNICEF report that globally 19.5 million infants missed the routine immunization services, and 60 % of these children reside in developing countries which include India, Pakistan, Indonesia, Angola, Brazil, the Democratic Republic of the Congo, Ethiopia, Iraq, Nigeria, and South Africa [3].

India’s immunization program dates back to 1978, when the Expanded Program of Immunization (EPI) was launched by the government of India [4]. When the Indian government launched Universal Immunization Programme (UIP) in 1985, it aimed to provide vaccination against six diseases: tuberculosis, diphtheria, pertussis, tetanus, polio, and measles [4]. Implementation of the government’s immunization program has helped reduce the diseases and the resultant deaths, but the progress is plodding [5]. As per the recent estimates from National Family Health Survey-2015-16 (NFHS 4), only 62 % of the children are fully immunized in India [6]. Previous studies have highlighted individual predictive factors for vaccination, including gender, age, birth order, and other household factors such as family size, number of children below age 3 years, household wealth, caste, and maternal education [7–9]. Studies indicate that the reasons associated with under-vaccination include the ones related to immunization systems, family characteristics, parental attitudes and knowledge, and limitations in immunization-related communication and information [10]. A study based on District Level Household Survey data concludes that after adjusting for various confounding factors like age, gender, state of residence and maternal education, other significant predictors of children’s vaccination status were religion, caste, birth order, place of delivery, number of antenatal care visits, and maternal tetanus vaccination [11]. Not only there is a slow rise in immunization coverage in India, but regional variations also exist [6, 12].

As a signatory of Sustainable Development Goal (SDG), India, like other countries, is pledged to secure healthy lives and promote well-being for all ages [13]. To achieve the health-related SDGs, one of the principal roles of the healthcare system is to provide equitable financing, which can protect people from experiencing financial hardship incurred due to the treatment of their illness [14].Compared to the developed countries covered by the tax-funded health system or social health insurance, developing countries depend on out-of-pocket spending on health, which drives them into the poverty cycle [15]. The high and increasing cost of health is one of the public health challenges the developing nations face, and India is not an exception to this [16]. Health system in India is characterized by the co-existence of public and private health centres. Health spending is consistently high among the poor, less educated, uninsured, rural households, female-headed households, households with members suffering from chronic illness, and households with older people [17]. Various world leaders highlighted the importance of healthcare payments as a cause of financial hardship and promoted measures against catastrophic health expenditure [18, 19]. Immunization programs should strive to provide quality services that are accessible, convenient, reliable, friendly, affordable, and acceptable [20]. On the other hand, recent literature suggests the importance of vaccination on the broader economy of the middle and low-income countries, stating that immunization programmes can reduce the proportion of households facing catastrophic out-of-pocket health expenses, mainly in lower socioeconomic groups [21]. Thus, vaccines could have an important role in poverty reduction.

Currently, India’s universal immunization programme covers a birth cohort of 26 million infants, making it the largest in the world. However, India lags behind its many less-developed neighbours in vaccination rates due to reasons which include a huge population with relatively high growth rate, geographical diversity and some hard to reach populations, lack of awareness regarding vaccination, inadequate delivery of health services, inadequate supervision, and monitoring, lack of micro-planning and general lack of inter-sectoral coordination, and weak VPD surveillance system [22]. The cost of delivering routine immunization varies widely across facilities within countries and across countries. India bears a total immunization expenditure cost of US$718 million in 2012–13 [23]. A study based on a random sample of 255 public health facilities from 24 districts across seven states—Bihar, Gujarat, Kerala, Meghalaya, Punjab, Uttar Pradesh, and West Bengal indicated there was wide variation in the weighted average state-level cost per dose delivered inclusive of vaccine costs (US$1.38 to US$2.93) and, for the cost per DPT3 vaccinated child (US$20.08 to US$34.81) [24]. These studies mostly talk about the government’s spending and budget allocation on immunization. However, individuals too bear expenditure on immunization, which is a not much-focussed area of research. Thus, the present paper attempts to check the individual expenditure on Immunization in India. In this context, this study tries to estimate the expenditure on Immunization which an individual bears and the factors affecting immunization coverage at the regional level. A study on expenditure incurred on Immunization could help in explaining the broader economic factors affecting the immunization coverage at the regional level and thus help understand the inequalities in child health status.

## Materials and methods

### Data

This study used the 75th round of schedule 25.0 data on key indicators of Household social consumption in India: health. The study used nationally representative cross-sectional data collected by the National Sample Survey Organisation (NSSO) during 2017-18.The first full-scale NSS health survey was conducted in the 28th round of NSS (1973- 74). Since the 1990 s, there were four health surveys of NSO (erstwhile NSSO): those of the 52nd round (July 1995-June 1996), the 60th round (January 2004-June 2004), the 71st round (January 2014-June 2014), and the 75th round (July 2017-June 2018). A detailed methodology of data collection and sampling design was published elsewhere [25, 26].

The objective of the 75th round survey was to generating basic quantitative information on the health sector [27]. The survey covered the whole of the Indian Union except the villages in the Andaman and Nicobar Islands, which were difficult to access. It collected data from 1,13,823 households spread over every district of the country. The survey adopted a stratified multi-stage sampling design to provide the prevalence rate at the state and national level of general morbidity by age-group and gender, as well as of specific categories of ailment [27]. The first stage units (FSU) are the Census villages (Panchayat wards for Kerala) in the rural sector and Urban Frame Survey (UFS) blocks in the urban sector. The ultimate stage units (USU) are households in both sectors. In large FSUs, one intermediate stage of sampling is the selection of two hamlet-groups (hgs)/ sub-blocks (sbs) from each rural/ urban FSU. Each district was a stratum. Within each district of a State/UT, two basic strata have been formed: (i) rural stratum comprising of all rural areas of the district and (ii) urban stratum comprising of all the urban areas of the district. For the rural sector, from each stratum/sub-stratum, required number of sample villages has been selected by Probability Proportional to Size With Replacement (PPSWR), size being the population of the village as per Census 2011. For the urban sector, from each stratum/sub-stratum, FSUs have been selected by Probability Proportional to Size With Replacement (PPSWR), size being the number of households of the UFS Block. Both rural and urban samples are drawn in the form of two independent sub-samples [27].

Further, the survey provides the estimates of children having received specific vaccination, of fully immunized, and children who had received no immunization, for appropriate age-groups of children aged 0–5 years [27] to generate SDG (Sustainable Development Goals) [28] indicators of immunization status. Expenditure on immunization, if any, during the last 365 days and status of immunization of children as on the date of the survey (age 0–5 years) was asked in block 10b of the schedule 25.0. The sample size for this study was 70,246 children age below 5 years.

### Outcome variables

Full immunization and expenditure on immunization were the two outcome variables for this study. According to the WHO guideline [29], “full immunization” coverage is defined as a child has received a BCG vaccination against tuberculosis; three doses of DPT vaccine to prevent diphtheria, pertussis, and tetanus (DPT); at least three doses of polio vaccine; and one dose of measles vaccine. For the analysis of the study, the variable on immunization had been categorized as if a child received all these vaccines it was coded ‘1’ and ‘0’ otherwise. Expenditure on immunization was a continuous variable and measured in rupees (Rs.). The question was asked to the respondents about expenditure on immunization, if any, during the last 365 days that is directly available in the data.

### Independent variables

Relevant predictors for immunization and expenditure on immunization included in this analysis were place of residence (urban, and rural), religion (Hindu, Muslim, and others), caste (Scheduled Caste, Scheduled Tribe, Other Backward Caste and Others), gender (male, and female), wealth quintile (poor, middle, and rich), place of immunization (public, and private), and region (North, Central, East, Northeast, West, and South). The wealth quintile generated using monthly per capita expenditure (MPCE) of the respective household [25, 26].The information on households’ usual monthly consumer expenditure (UMCE) was collected through a single question in the survey. To calculate the MPCE, UMCE has been divided by household size.

## Methods

Descriptive statistics and multivariate regression analysis [30] were used to understand the predictors for full immunization among children in India. Further, the two-part model had been employed to inspect the determinants of expenditure on immunization and adjusted expenditure for immunization by socio-economic characteristics of the household. The two-part model was used as the expenditure for immunization data had skewed distribution, and 89 % of households did not incur expenditure for immunization (zero values). Endogeneity bias was checked before running the two-part model inclusive of all independent variables including place of immunization.

The two-part model separates the decision-making process into two steps [31, 32]. In the first step, the probability of a household to incur expenditure on immunization was modelled using a logit model. In the second step, the expenditure on immunization was estimated using Ordinary Least Square (OLS) regression. The dependent variable was in the binary form where ‘0’ represented those who did not incur any expenditure on immunization, and ‘1’ represented those who had incurred some expenditure on immunization. Given any positive expenditure on immunization, the second step estimated the intensity of expenditure on immunization using an OLS regression, where the dependent variable was the log of expenditure on immunization.

A two-part model is a robust statistical model needed to handle with a small number of dependent variables [33]. These variables are distinguished by the fact that the range of values they can assume has a lower bound that occurs in a significant number of observations. The following is the basic framework. Assume that there is an event that could or could not happen. When this happens, a positive random variable is seen. When it doesn’t, the observed result is set to zero, resulting in a zero-censored variable [33].The event is represented by a specific condition in explaining individual yearly health spending, for example. If the sickness arises, some non-free therapy will be required, resulting in a positive expenditure [33]. A two-part model allows the filtering mechanism and the outcome to be represented separately in these scenarios. As a specific sort of mixture model, it allows the zeros and non-zeros to be created by varied densities. The zeros are typically handled using a model for the probability of a positive outcome,

$$\varnothing \left(y>0\right)=Pr\left(y>0|x\right)=F\left(x\delta \right)$$

Where **x** is a vector of explanatory variables, ***δ*** is the corresponding vector of parameters to be estimated, and *F* is the cumulative distribution function of an independent and identically distributed error term, typically chosen to be from extreme value (logit) or normal (probit) distributions. For the positives, the model is usually represented as

$$\varnothing \left(y|y>0, x\right)=g\left(x\gamma \right)$$

Where **x** is a vector of explanatory variables, ***γ*** is the corresponding vector of parameters to be estimated, and *g* is an appropriate density function for *y|y >* 0. The likelihood contribution for an observation can be written as,

$$\varnothing \left(y\right)={\left\{1-F\left(x\delta \right)\right\}}^{i\left(i=0\right)}*{\left\{F\left(x\delta \right)g\left(x\gamma \right)\right\}}^{i\left(y>0\right)}$$

Where *i*(.) denotes the indicator function. Then, the log-likelihood contribution is

$$ln\left\{\varnothing \left(y\right)\right\}=i\left(i=0\right)ln\left\{1-F\left(x\delta \right)\right\}+i\left(i=0\right)\left[ln\left\{F\left(x\delta \right)\right\}+ln\left\{g\left(x\gamma \right)\right\}\right]$$

Because the ***δ*** and ***γ*** parameters are additively separable in the log-likelihood contribution for each observation, the models for the zeros and the positives can be estimated separately [33].

## Results

**Fig. 1:**
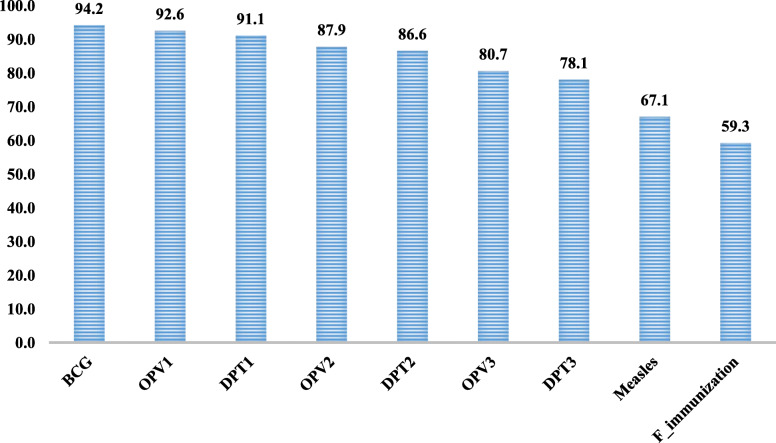
Immunization status among children under 5 years in India, 2017-18. Note: BCG: Bacille Calmette-Guerin; OPV: Oral Poliovirus Vaccines; DPT: Diphtheria-Pertussis-Tetanus

The immunization status among children aged 0-5- years is shown in Fig. 1. About 94.2 % of children aged 0–5 years received the BCG vaccine, 92.6 % of the children received the OPV1 vaccine, and 91.1 % received the DPT 1 vaccine. The percentage of children receiving the OPV 2 vaccine dropped to 87.9 %, further dropping to 80.7 % for OPV3 vaccination. Nearly 86.6 % of the children received DPT 2 vaccination which further reduced to 78.1 % for DPT3 vaccine. About 67.1 % of children received measles vaccination. The overall prevalence of full immunization as per the 75th round of NSSO data was 59.3 %.

Table 1 represents the descriptive statistics of the study population. It was found that 25.4 % of the study population was from an urban place of residence. About 17.7 % of the respondents were from the Muslim religion. About 10.2 and 22 % of the respondents were from the Scheduled Tribes and Scheduled Caste category, respectively. About 48.3 % of the children were female. Nearly 23.3 % of the respondents were from the rich wealth quintile. About 5.6 % of the children got immunized at the private facility. About 27.5 % of the respondents were from central region of India.

**Table 1:** Descriptive summary of the study population

Background characteristics	Percentage	Sample
**Place of residence**		
Rural	74.6	43,217
Urban	25.4	27,029
**Religion**		
Hindu	78.4	51,289
Muslim	17.7	11,755
Others	3.9	7,202
**Caste**		
Schedule Tribe	10.2	10,346
Schedule Caste	22.0	12,904
Other Backward Class	45.2	28,543
Others	22.6	18,453
**Sex**		
Male	51.7	36,229
Female	48.3	34,017
**Wealth quintile**		
Poor	43.2	23,878
Middle	33.5	23,141
Rich	23.3	23,227
**Place of immunization**		
Public	94.4	64,489
Private	5.6	5,626
**Region**		
North	15.2	13,370
Central	27.5	14,026
East	24.0	12,431
Northeast	3.3	8,824
West	13.2	8,332
South	17.0	13,263
**Total**	100.0	70,246

The percentage distribution and mean expenditure of full immunization among children aged 0–5 years were depicted in Table 2. Table 1 revealed that almost 95 % of the children (94.4 %) were vaccinated at public facilities, and the remaining 5.6 % were vaccinated at private facilities. In continuation of that, results from Table 2 noted that almost three-fifths (60.6 %) of the children, who were vaccinated at public health facilities (94.4 %), were fully immunized at the public facility. A similar interpretation can be given for the vaccination of children in private facilities. Around 5.6 % of children who were immunized at private facilities, of them almost 40.4 % were fully immunized at private facilities. Full immunization was highest in Manipur (75.2 %) and lowest in Nagaland (12.8 %). States like Uttarakhand, Himachal Pradesh, Haryana, Kerala, Mizoram, and Andhra Pradesh had over 70 % of their children fully immunized. On the contrary, states like Pondicherry, Tripura, Arunachal Pradesh, Assam, Bihar, and Delhi had less than 50 % of their children fully immunized. The mean expenditure incurred on immunization varies from as low as Rs. 33 in Tripura to as high as Rs. 1009 in Delhi. On average, the country incurs an expenditure of Rs266 for full immunization. States like Meghalaya, Tamil Nadu, Karnataka, Goa, and Maharashtra incur a mean expenditure of more than Rs 400. While states like Manipur, Sikkim, and Assam incur a mean expenditure of less than Rs 100. The percentage distribution of full immunization at a public facility and private facility was also presented in the table. The full immunization received at a public facility was lowest in Nagaland (13.3 %) and highest in Manipur (76.1 %). The mean expenditure incurred on full immunization at a public facility varies from less than Rs 3 in Lakshadweep to Rs 200 in Meghalaya. The mean expenditure incurred on full immunization at a public facility is more than Rs 50 in states like Orissa, Jammu and Kashmir, Nagaland, Telangana, and Arunachal Pradesh. Mean expenditure was less than Rs 10 in states like Rajasthan, Chhattisgarh, and Delhi. About 66.6 % of children in Telangana receive full immunization at a private facility. Full immunization at private facilities was high in states like Karnataka, Orissa, Chhattisgarh, Kerala, and Haryana, where more than 50 % of the children receive immunization at private hospitals/facilities. The mean expenditure incurred on immunization at a private facility was highest in Delhi (Rs 4274), followed by West Bengal (Rs 4031). States like Andhra Pradesh, Himachal Pradesh, Jammu and Kashmir, Goa, and Madhya Pradesh incur a mean expenditure of over Rs 3000 for immunization at a private facility. In Tripura, where only 0.4 % of the children were immunized at a private facility, incurred a mean expenditure of Rs 186 in the private facility. States like Nagaland and Sikkim, where the percentage of children immunized at a private facility was about 6 and 3 %, incurs an expenditure of about Rs 412 and Rs 920, respectively. Figure-S1 in the supplementary file represents the maps for expenditure on immunization in 88 regions of India.

**Table 2:** Percentage distribution and mean expenditure of full immunization among children under 5 years in states of India, 2017-18

States	Total		Public		Private	
	**Full immunization**	**Mean expenditure of immunization (in Rupees)**	**Full immunization**	**Mean expenditure of immunization (in Rupees)**	**Full immunization**	**Mean expenditure of immunization (in Rupees)**
Jammu & Kashmir	64.5	256	65.1	61	47.1	3133
Himachal Pradesh	72.0	130	73.1	26	41.7	3163
Punjab	61.7	317	64.2	20	30.8	2274
Chandigarh	64.9	295	66.3	11	22.1	2694
Uttarakhand	70.5	148	71.5	25	18.2	1710
Haryana	72.1	382	73.2	13	61.9	2873
Delhi	47.9	1009	49.2	9	19.4	4274
Rajasthan	57.3	118	58.8	4	22.9	1709
Uttar Pradesh	54.6	174	55.8	18	36.2	1667
Bihar	48.4	184	49.2	32	24.4	2084
Sikkim	65.2	63	65.9	32	3.3	920
Arunachal Pradesh	41.3	237	43.1	184	8.9	1403
Nagaland	12.8	101	13.3	68	6.3	412
Manipur	75.2	49	76.1	18	43.4	822
Mizoram	73.4	103	73.6	39	38.7	1493
Tripura	40.2	33	42.8	27	0.4	186
Meghalaya	52.0	432	53.5	200	19.5	1619
Assam	46.4	93	46.8	31	28.5	1453
West Bengal	66.3	355	67.7	21	33.7	4031
Jharkhand	58.4	119	59.9	16	16.8	2066
Orissa	68.0	146	68.2	51	51.4	2445
Chhattisgarh	65.2	150	65.4	6	55.5	2606
Madhya Pradesh	62.9	188	64.5	11	20	3010
Gujarat	59.6	356	61.1	25	34.5	2099
Daman & Diu^a^	45.3	162	46.8	8	0	2220
Dadra & Nagar Haveli	62.0	293	61.3	17	72.4	3550
Maharashtra	58.6	479	60.1	15	46.1	2470
Andhra Pradesh	73.6	306	74.1	30	49.2	3828
Karnataka	61.8	461	62.7	49	50.3	1858
Goa	59.7	461	59.9	26	46.2	3071
Lakshadweep	70.2	116	70.6	3	7.7	1200
Kerala	72.8	331	73.9	31	60.5	1439
Tamil Nadu	57.5	449	58.8	22	47.3	2026
Pondicherry	34.1	153	34.2	4	30.9	1873
Andaman & Nicobar Island	63.3	272	65.1	62	10.8	2721
Telangana	70.1	381	70.5	95	66.6	2399
**Total**	**59.32**	**266**	**60.6**	**29**	**40.4**	**2248**

^a^In Daman and due full Immunization in 0 % but there is expenditure shown. That is because some expenditure was incurred on partial Immunization too; 1USD = 74.12 Rupees

The percentage distribution and odds ratio of full immunization by background characteristics among children aged 0–5 years is presented in Table 3. About 58.5 % of the children in rural areas and 61.7 % of children in urban areas were fully immunized. Nearly 59.1 % of Hindu children, 59.8 % of Muslim children, and 61.1 % of children belonging to other religion were fully immunized. About 59.7 %, 58.4 %, 59.2 %, and 60.3 % of the children belonging to ST, SC, OBC, and others category were fully immunized, respectively. About 58.6 % of male and 60.1 % of female children were fully immunized. Almost 56.9 of children who belonged to the poor wealth quintile were fully immunized, while 62.6 % of the children belonging to the rich quintile were fully immunized. About 60.6 % of the children were immunized at public health care facilities, while 40.4 % of the children were immunized at private health facilities. Immunization was highest in the Southern region of the country, where 65.9 % of children were fully immunized. Immunization among children was lowest in the north-eastern region, where 48.4 % of children were fully immunized.

**Table 3:** Percentage distribution and odds ratio for full immunization by background characteristics among children under 5 years in India, 2017-18

Background characteristics	Percentage distribution	OR [95 % Conf. Interval]
**Place of residence**		
Rural	58.5	Ref.
Urban	61.7	1.04***(1.035–1.037)
**Religion**		
Hindu	59.1	Ref.
Muslim	59.8	1.05***(1.049–1.051)
Others	61.1	1.09***(1.084–1.089)
**Caste**		
Schedule Tribe	59.7	Ref.
Schedule Caste	58.4	0.88***(0.882–0.885)
Other Backward Class	59.2	0.88***(0.876–0.879)
Others	60.3	0.93***(0.928–0.931)
**Sex**		
Male	58.6	Ref.
Female	60.1	1.06***(1.064–1.065)
**Wealth quintile**		
Poor	56.9	Ref.
Middle	60.2	1.11***(1.104–1.106)
Rich	62.6	1.14***(1.134–1.137)
**Place of immunisation**		
Public	60.5	Ref.
Private	40.4	0.39***(0.389–0.391)
**Region**		
North	61.1	Ref.
Central	57.4	0.91***(0.91–0.913)
East	57.5	0.91***(0.906–0.909)
Northeast	48.4	0.59***(0.585–0.588)
West	58.9	0.91***(0.907–0.91)
South	65.9	1.25***(1.244–1.248)

*Ref* Reference

*p < 0.1;**p < 0.05 and ***p < 0.01

The logistic regression results reporting the odds ratio based on background characteristics indicate that in urban areas, the children were 4 % significantly more likely to be fully immunized than their rural counterparts. Children from other religious groups were 9 % significantly more likely to be fully immunized than children from the Hindu religion. The children from Scheduled Castes had 12 % significantly lower likelihood of being immunized than children from Scheduled Tribe. Female children were 6 % significantly more likely to be fully immunized as compared to male children. Children from the rich wealth quintile were 14 % significantly more likely to be fully immunized than children from the poor wealth quintile. Children were 61 % significantly more likely to get immunized at a public facility in reference to a private facility. Children from the southern region of India were 25 % significantly more likely to get immunized in reference to children from the northern region of India.

Table 4 presents the mean expenditure (in Rs) on immunization among children aged 0–5 years by their background characteristics. The mean expenditure in rural areas was Rs. 85.While in urban areas, it was Rs. 544. The mean expenditure incurred by the Hindu (Rs. 289) and other (Rs. 289) religion was almost the same while that of the Muslims (Rs. 151) was less. The mean expenditure on immunization was highest for children belonging to other (Rs. 523) social category followed by OBC (Rs. 220), ST (Rs. 103) and SC (Rs. 103). The mean expenditure on immunization was almost equal for male and female children and was around Rs. 266. The poor have a mean expenditure of Rs. 55 on immunization, while the rich had a mean expenditure of Rs. 618. The expenditure on immunization was more in the case of private centre (Rs. 2248) than in public facilities (Rs. 29). The mean expenditure on immunization was highest in the western region (Rs. 431), followed by the southern region (Rs. 388). It was less in the north-eastern region where they incur a mean expenditure of Rs. 99 on immunization.

**Table 4:** Mean expenditure on immunization among children under 5 years by background characteristics  in India, 2017-18

Background characteristics	Mean Exp. (in Rupees)	95 % Conf. Interval	
**Place of residence**			
Rural	85	79	90
Urban	544	516	572
**Religion**			
Hindu	289	274	303
Muslim	151	132	171
Others	289	254	323
**Caste**			
Schedule Tribe	103	87	119
Schedule Caste	103	88	118
Other Backward Class	220	204	235
Others	523	490	557
**Sex**			
Male	266	250	282
Female	266	249	283
**Wealth quintile**			
Poor	55	49	61
Middle	115	104	126
Rich	618	587	650
**Place of immunisation**			
Public	29	27	30
Private	2248	2155	2341
**Region**			
North	277	249	304
Central	175	153	196
East	212	185	240
Northeast	113	99	127
West	431	388	475
South	388	357	419

1USD = 74.12 Rupees

In Table 5, we have estimated the expenditure by socio-economic characteristics by using the two-part model. Results suggest that the probability of incurring expenditure on Immunization for children was high among children in urban areas in comparison to rural areas (β = 0.22, *p* < 0.05). Gender differentials can be well observed, i.e., female children had a lower likelihood to incur expenditure for immunization in reference to male children (β=-0.09, *p* > 0.05). Children from the richest wealth quintile had a higher likelihood of incurring expenditure on Immunization in reference to children from the poor wealth quintile ((β = 0.35; *p* < 0.05). Private health facilities had more likelihood of incurring expenditure on Immunization among children in India in reference to the public facility (β = 4.02; *p* < 0.05). It was further found that the children from the north-eastern region had a higher likelihood of incurring expenditure on Immunization in reference to children from the northern region (β = 1.5; *p* < 0.05).

**Table 5:** Predicted mean health expenditure (in rupees) by socio-economic and demographic correlates for children under 5 years in India, 2017-18

Background characteristics	Logit coef. (95 % CI)	Regress log coef. (95 % CI)	Predicted mean health expenditure (in Rupees)
**Place of residence**			
Rural	Ref.	Ref.	58
Urban	0.22***(0.219–0.224)	0.25***(0.246–0.25)	273
**Religion**			
Hindu	Ref.	Ref.	112
Muslim	-0.23***(-0.235 - -0.229)	-0.18***(-0.186 - -0.182)	99
Others	0.34***(0.333–0.342)	0.03***(0.025–0.032)	182
**Caste**			
Schedule Tribe	Ref.	Ref.	47
Schedule Caste	0.42***(0.412–0.421)	-0.08***(-0.085 - -0.077)	57
Other Backward Class	0.70***(0.7–0.708)	-0.09***(-0.09 - -0.081)	104
Others	0.60***(0.597–0.606)	0.04***(0.032–0.04)	212
**Sex**			
Male	Ref.	Ref.	121
Female	-0.09***(-0.093 - -0.089)	-0.13***(-0.136 - -0.133)	103
**Wealth quintile**			
Poor	Ref.	Ref.	49
Middle	-0.08***(-0.084 - -0.079)	0.04***(0.037–0.042)	66
Rich	0.35***(0.348–0.354)	0.29***(0.285–0.291)	297
**Place of immunisation**			
Public	Ref.	Ref.	21
Private	4.02***(4.016–4.022)	1.57***(1.569–1.573)	1656
**Region**			
North	Ref.	Ref.	101
Central	0.35***(0.35–0.357)	-0.35***(-0.35 - -0.344)	69
East	0.44***(0.432–0.44)	0.08***(0.077–0.084)	81
Northeast	1.5***(1.496–1.506)	-0.5***(-0.507 - -0.497)	71
West	0.45***(0.446–0.453)	-0.08***(-0.08 - -0.073)	199
South	0.17***(0.162–0.169)	-0.08***(-0.086 - -0.08)	177

1USD = 74.12 Rupees

*Ref:* Reference

*p < 0.1;**p < 0.05 and ***p < 0.01

In Table 5, we have also presented the adjusted expenditure on immunization among children by socio-economic characteristics in India. The expenditure on mmunization was almost 4.7 times higher for children who belong to urban areas than in rural areas. Children who belong to other religious categories were spending almost 1.62 times higher the expenditure on immunization than children who belong to the Hindu religion. The adjusted expenditure on immunization was almost six times higher for the children who belong to the richer wealth quintile (Rs. 297 vs. Rs. 49) in comparison to children who belong to the poor wealth quintile. Expenditure on immunization was higher for male children compared to female children. Expenditure on immunization was multiple times higher when children were immunized in private facilities (Rs. 1656 vs. Rs. 21) than in public health care facilities. When predicted expenditure on immunization was observed for six regions of India, it was found that the west region was having the highest OOPE for immunization among children in India.

## Discussion

Despite launching the Universal Immunization Programme (UIP) in 1985, immunization coverage has progressed slowly and is far from the desired goals [22, 34, 35]. To tackle immunization, Mission Indradhanush was launched by the Government of India in 2014 to increase full immunization coverage to 90 % in India. Even after launching Mission Indradhanush, the country’s average immunization coverage remains below par [36].

### Inequalities in Immunization by region and by various background characteristics

Around 60 % of the children have received full immunization. For OPV1, approximately 93 % of children were immunized, which declined from 88 % for OPV2 to 81 % for OPV3. Similarly, the same trend in dropout has been seen for the DPT also. Itimi et al. (2012), in their study carried out in rural-urban set up in Nigeria, also found a dropout in DPT [37].They concluded that the dropout occurs due to lack of motivation among the children’s parents, relocation of the service centres, absence of vaccinator, non-availability of vaccines, and the malicious rumours about the immunization. Usman et al. (2010), in their cohort study involving 366 mother-infant pairs from six rural immunization centres around Karachi, Pakistan, found that children who received DPT dose 1 in a timely manner and lived closer to the immunization site were more likely to receive the subsequent doses [38]. Randomized controlled trials in Pakistan demonstrated that providing mothers with a redesigned immunization card and home- or centre-based education on the importance of vaccines help improve the DPT3 completion rate [39, 40]. Our study found that of all four vaccines, the immunization for measles vaccine is the least. It has been pointed out that measles vaccination has been poorly addressed in India [34].

In Nagaland, Tripura, Arunachal Pradesh, Assam, and Bihar, not even half of the children were fully immunized. A study carried out in BIMARU (Bihar, Madhya Pradesh, Rajasthan, and Uttar Pradesh) states found that in these states, the full immunization is lower than the national average [41]. In Manipur, Andhra Pradesh, Mizoram, Kerala, Haryana, Himachal Pradesh, and Uttarakhand, more than 70 % of children were fully immunized. The north-eastern states showed a contrast difference. Nagaland has the lowest full immunization coverage, whereas Manipur has the highest full immunization coverage. Lalneizo & Reddy (2010), in their study carried out in North-eastern states, found that Nagaland had the lowest level of full immunization among the children, and states like Manipur and Mizoram had an adequate level of full immunization coverage [42]. They believe that the deviation across the North-eastern states raises further questions, which require in-depth micro-level studies to answer these queries. The coverage of full immunization has improved significantly in all the states of India; the North-South divide is still a gap to overcome [43].

A higher proportion of children were fully immunized in urban areas than in rural areas. In India [8, 12] and around the world [44], it has been found that immunization coverage is higher in urban areas than in rural areas. Pande and Yazbeck [12] cited various plausible reasons for higher immunization coverage in urban areas as against rural areas; one such reason is demand failure where rural people may not demand or may not use available immunization services; families in rural areas may not be adequately informed about the immunization. The study highlighted wealth as one of the strongest predictors of inequality for immunization. Previous studies in various settings have also found that the accumulation of wealth within a household improves immunization rates among children [8, 12, 45].

### Inequalities in expenditure for immunization

Through the last decade, India has made remarkable progress in wide-spreading immunization. The full immunization has improved significantly over time, but with inequity at every level [46]. As discussed in the above section, immunization depends on various background characteristics. In this section, we have tried to explore the interplay between expenditure and immunization.

The mean expenditure on immunization is higher in urban areas than in rural areas. Previous studies also highlighted that the expenditure is generally higher in urban areas than in rural areas [47]. The mean expenditure is higher in urban areas because of some plausible explanations; the first is the quality of supply in urban areas. Studies have identified various supply [12] and demand-side [48] factors that act as a barrier to the utilization of healthcare services. Supply-side arguments can be summed up by stating that rural residents do not have access to the same level of health services as their counterparts [49]. The second explanation is the inequality in the rural-urban income gap. Urban people tend to earn more than rural people; Not only in India [50, 51], the rural-urban income gap is highly persistent in other countries too [52, 53]. It is also possible that children in urban areas are being immunized at private facilities where the cost of immunization is higher. The mean expenditure of immunization is lesser in public hospitals than in private hospitals. Whatever amount people are spending for immunization in public hospitals can be attributed to the transportation cost or any other cost, as immunization in public hospitals is free of cost in India [34].

Religion is also one of the predictors of immunization. The result from the two-part model concludes that Muslim children are negatively associated with immunization compared to Hindu children. Not only religion but other factors like caste, gender, and wealth also seem to affect immunization. Social inequities in immunization coverage by gender, wealth, caste, and religion are well-documented [12].

Unlike previous studies [12, 54–56], this study noted a higher likelihood of immunization for female children than for male children. In contrast, the predicted mean health expenditure on immunization was lower among female children than in male children. The finding of higher immunization among female children needs elaborate discussion, and in light of the unavailability of similar results, it is pretty challenging to explain this finding. Further research is needed to explore the in-depth reasons for such findings. Perhaps, nationwide social welfare programmes like Integrated Child Development Services (ICDS) and Intensified Mission Indradhanush (IMI) succeeded in bringing awareness to everyone by setting up Anganwadi centers, a community-based service- delivery division of ICDS further leading to improved immunization among female children.

There are a few limitations in data. Firstly, data give the expenditure in totality rather than segregating it in various compounds like expenditure on travel, expenditure on vaccines, expenditure on doctor’s fee, etc. Secondly, the expenditure on immunization was self-reported. Self-reporting information can present bias as it is based on the individual’s recall capacity, leading to underestimation or overestimation of the prevalence of vaccination coverage [57, 58]. It is assumed that if the woman does not report the exact number of doses of Polio or DPT correctly, the estimates on full immunization are likely to be affected [59]. Thirdly, many covariates like mother’s education, occupation and childbirth order, etc., were not available in the data. Even after having some severe limitations, this study provides a broad picture of the prevalence of immunization and expenditure incurred on immunization among children in India.

## Conclusions

Based on the findings, we suggest looking for the supply and demand side for immunization as the dropout is still high for OPV and DPT. From the supply side, follow-up needs to be strengthened for OPV and DPT. The demand shall be created with the help of communication and mass media exposure. The government shall extensively promote the idea of using immunization through mass media, specifically in rural areas. Mobilizing village networks may also bring a significant decline in immunization dropouts. There is also a need to strengthen vaccine management to streamline vaccine supply and overcome gender bias in immunization. The scope of the present study is somewhat limited. It does not directly address why there is expenditure occurring on immunization in public hospitals when it is free of cost.

## Supplementary Information

**Additional file 1: Figure S1.** Maps for expenditure on Immunization in 88 regions of India

## Data Availability

The study utilizes a secondary source of data that is freely available in the public domain through, http://mospi.nic.in/unit-level-data-report-nss-75th-round-july-2017-june-2018-schedule-250social-consumption-health.

## Abbreviations

UIP: Universal Immunization Program
NSSO: National Sample Survey Office
WHO: World Health Organization
EPI: Expanded Program of Immunization
DPT: Diphtheria-Pertussis-Tetanus
MCV: Measles-Containing Vaccine
BCG: Bacillus Calmette-Guerin
NFHS: National Family Health Survey
NRHM: National Rural Health Mission
MI: Mission Indradhanush
SDG: Sustainable Development Goal
OPV: Oral Poliovirus Vaccines
OLS: Ordinary Least Square
SC: Scheduled Caste
ST: Scheduled Tribe
OBC: Other Backward Class
OOPE: Out-of-pocket expenditure
