# Supplementary material for: Household expenditure for immunization among children in India: a two-part model approach
Source: BMC Health Serv Res. 2021 Sep 22;21:1001. doi: 10.1186/s12913-021-07011-0 (PMC8459463; doi:10.1186/s12913-021-07011-0)
Supplement: Supplementary file 1 — Additional file 1: Figure S1. Maps for expenditure on Immunization in 88 regions of India [file 12913_2021_7011_MOESM1_ESM.docx]

**Appendix-1**

| 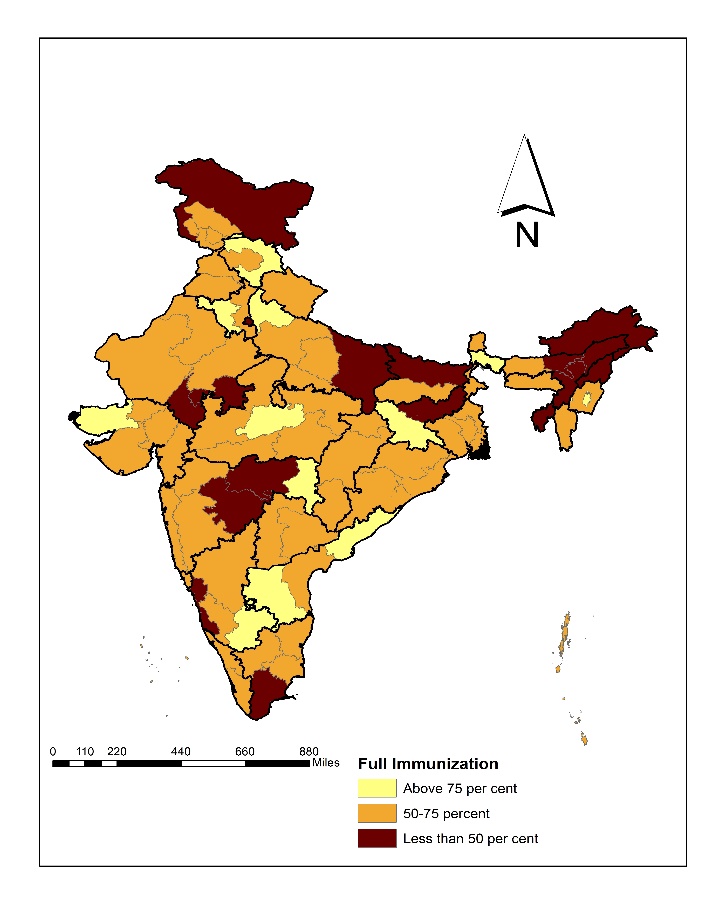 | 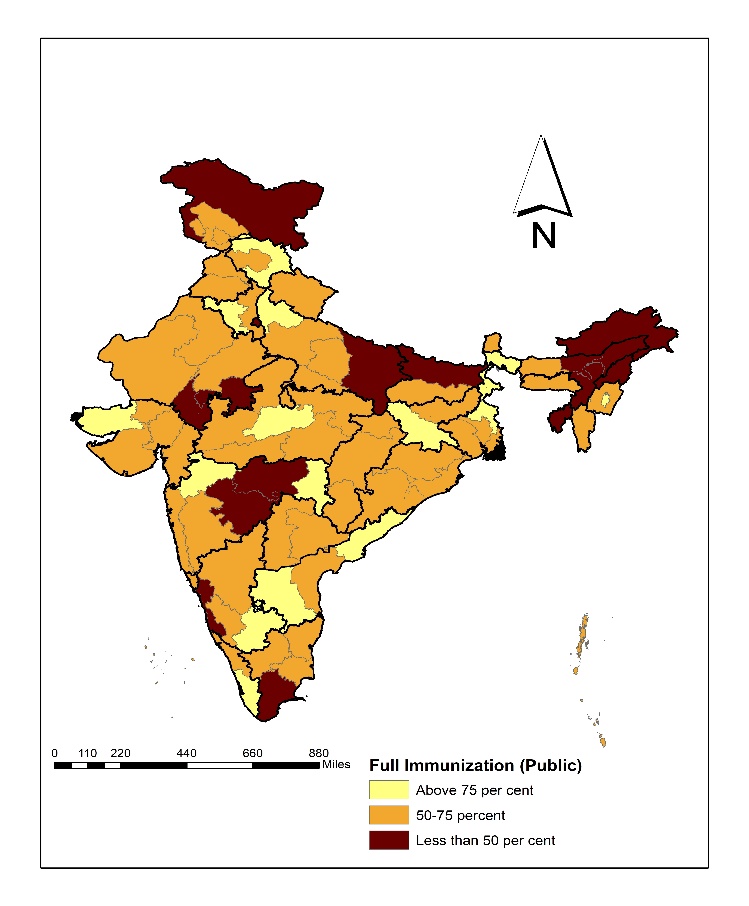 | 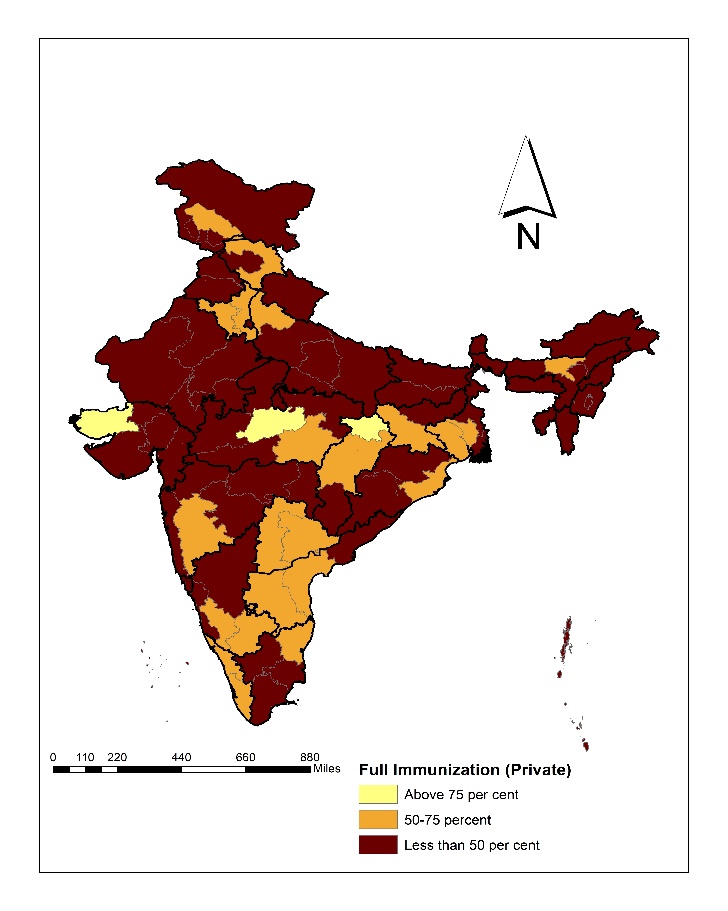 |
| --- | --- | --- |
| **Full Immunization in 88 regions of India (NSSO 75^th^ round.** | **Full Immunization in public facility in 88 regions of India (NSSO 75^th^ round.** | **Full Immunization in private facility in 88 regions of India (NSSO 75^th^ round.** |
| 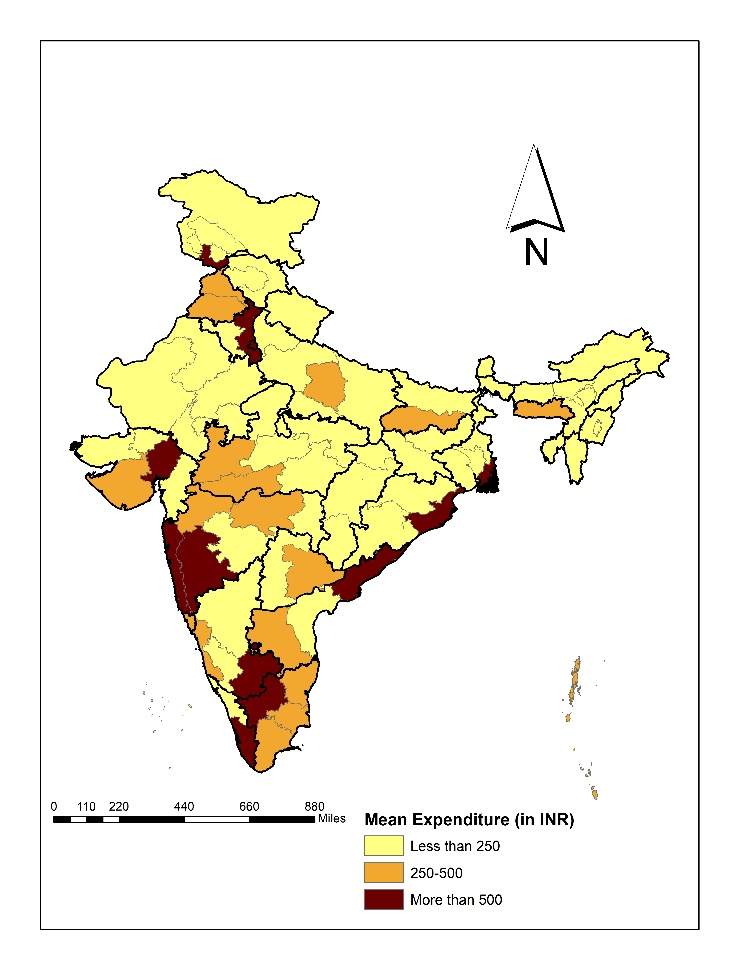 | 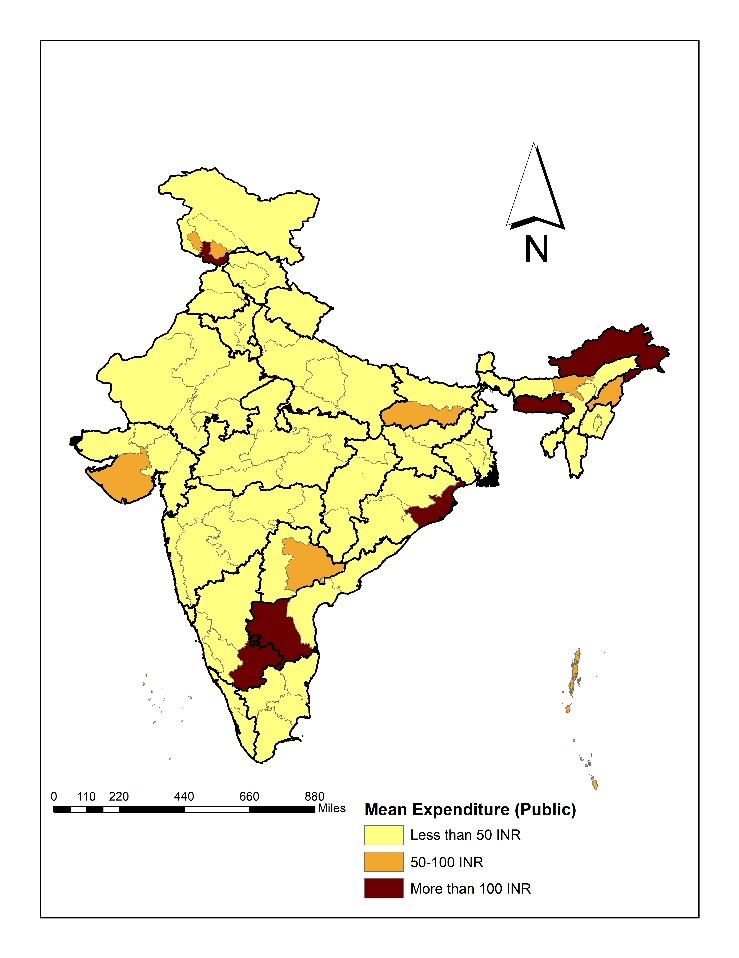 | 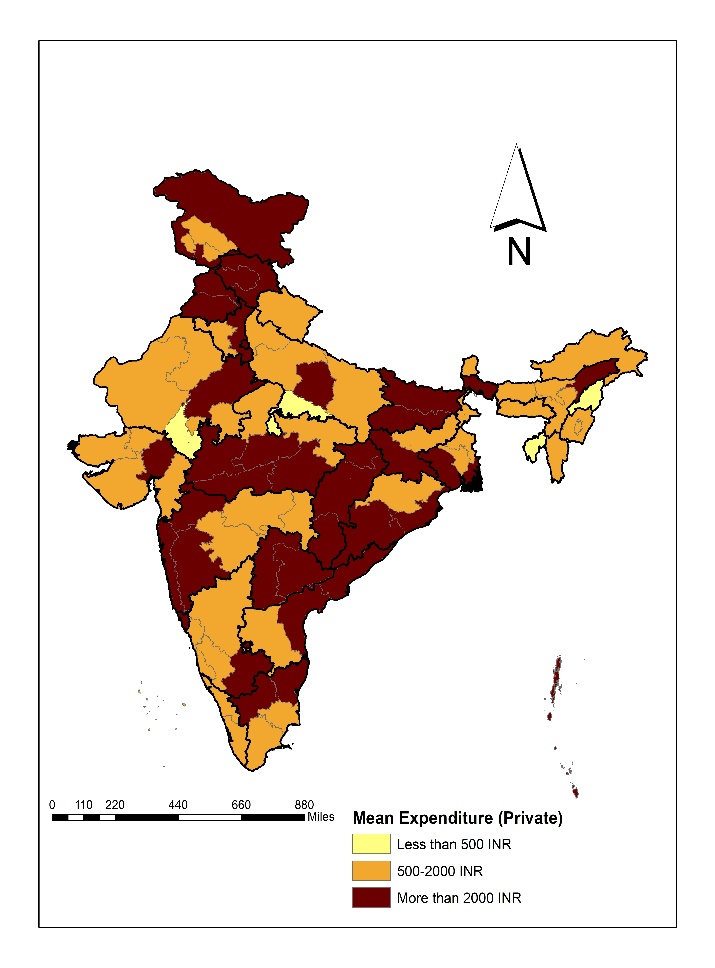 |
| **Mean Expenditure on Immunization in 88 regions of India (NSSO 75^th^ Round)** | **Mean Expenditure on Immunization in public facilty in 88 regions of India (NSSO 75^th^ Round)** | **Mean Expenditure on Immunization in Private facilty in 88 regions of India (NSSO 75^th^ Round)** |

**Figure-S1**. Maps for expenditure on Immunization in 88 regions of India
